# Supplementary figures and images for: Anti-GRP-R monoclonal antibody antitumor therapy against neuroblastoma
Source: PLoS One. 2022 Dec 16;17(12):e0277956. doi: 10.1371/journal.pone.0277956 (PMC9757561; doi:10.1371/journal.pone.0277956)

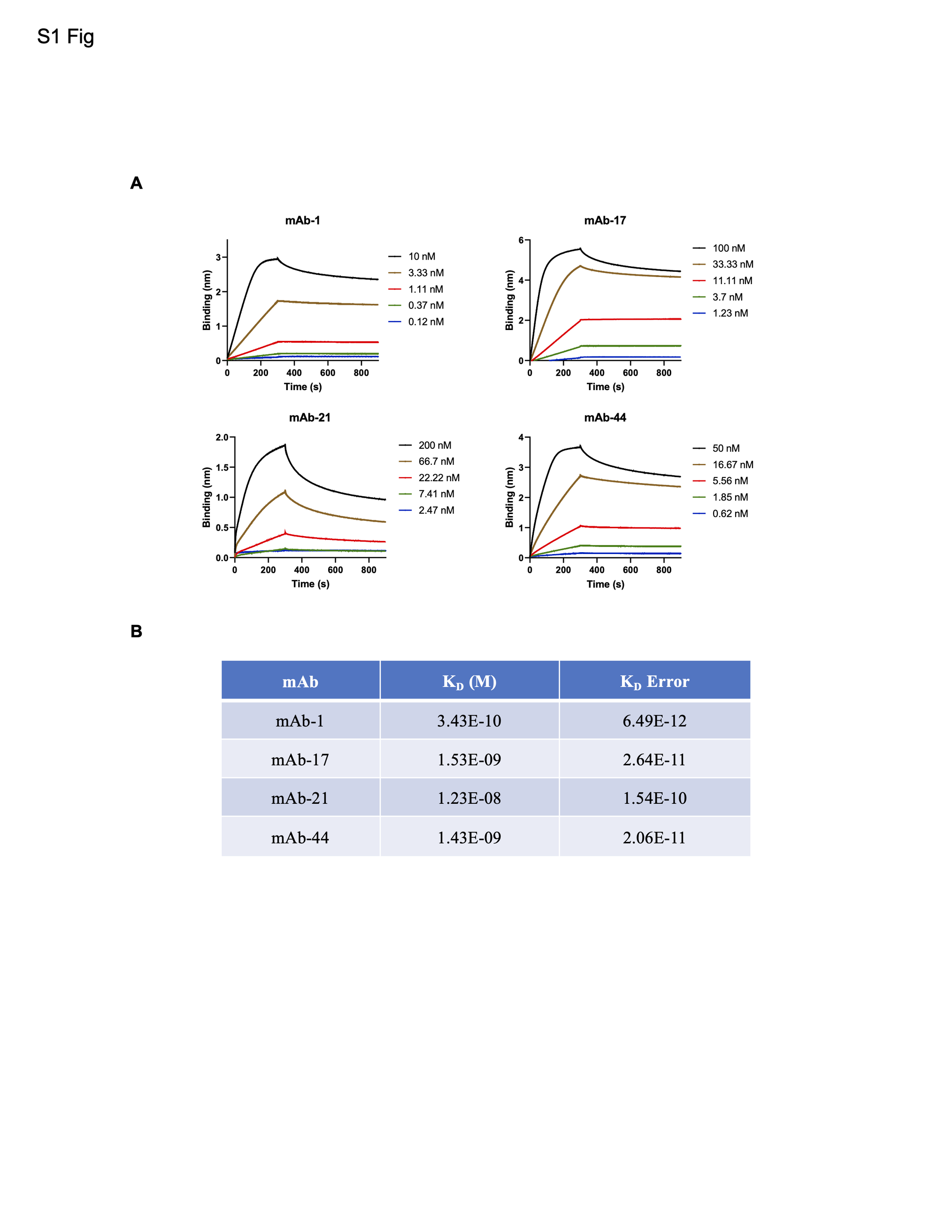

Supplement: S1 Fig — The KD value of GRP-R mAbs was measured using the 8-channel Octet RED96 system. The kinetic buffer without antibody was used as correction. The KD values were obtained by fitting the data to a 1:1 binding model. (TIF) [file pone.0277956.s001.tif]

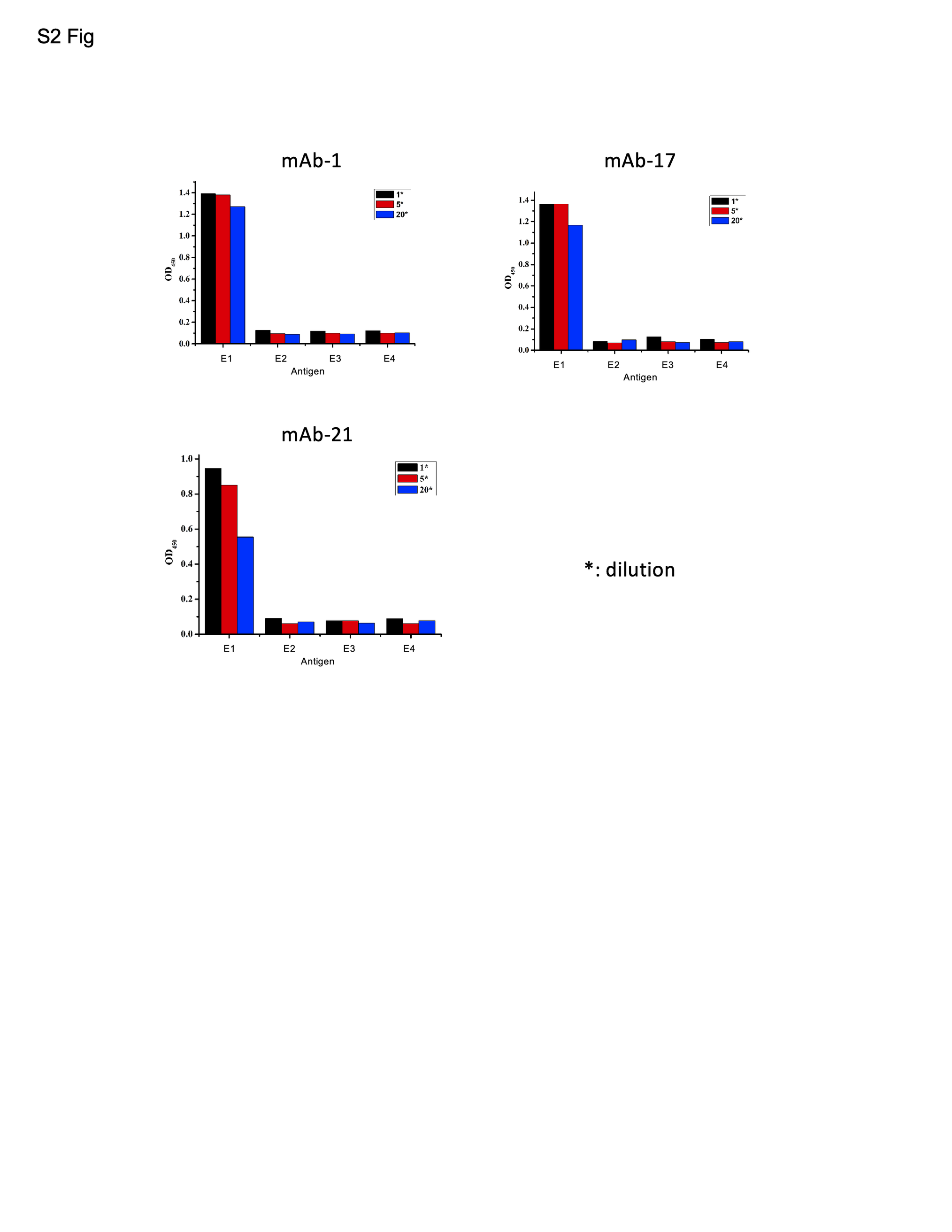

Supplement: S2 Fig — GRP-R mAbs cross binding test using ELISA coated with four different GRP-R extracellular domains E1, E2, E3, and E4. GRP-R mAb-1, mAb-17, and mAb-21 have no cross binding with GRP-R extracellular domain E2, E3, and E4. (TIF) [file pone.0277956.s002.tif]

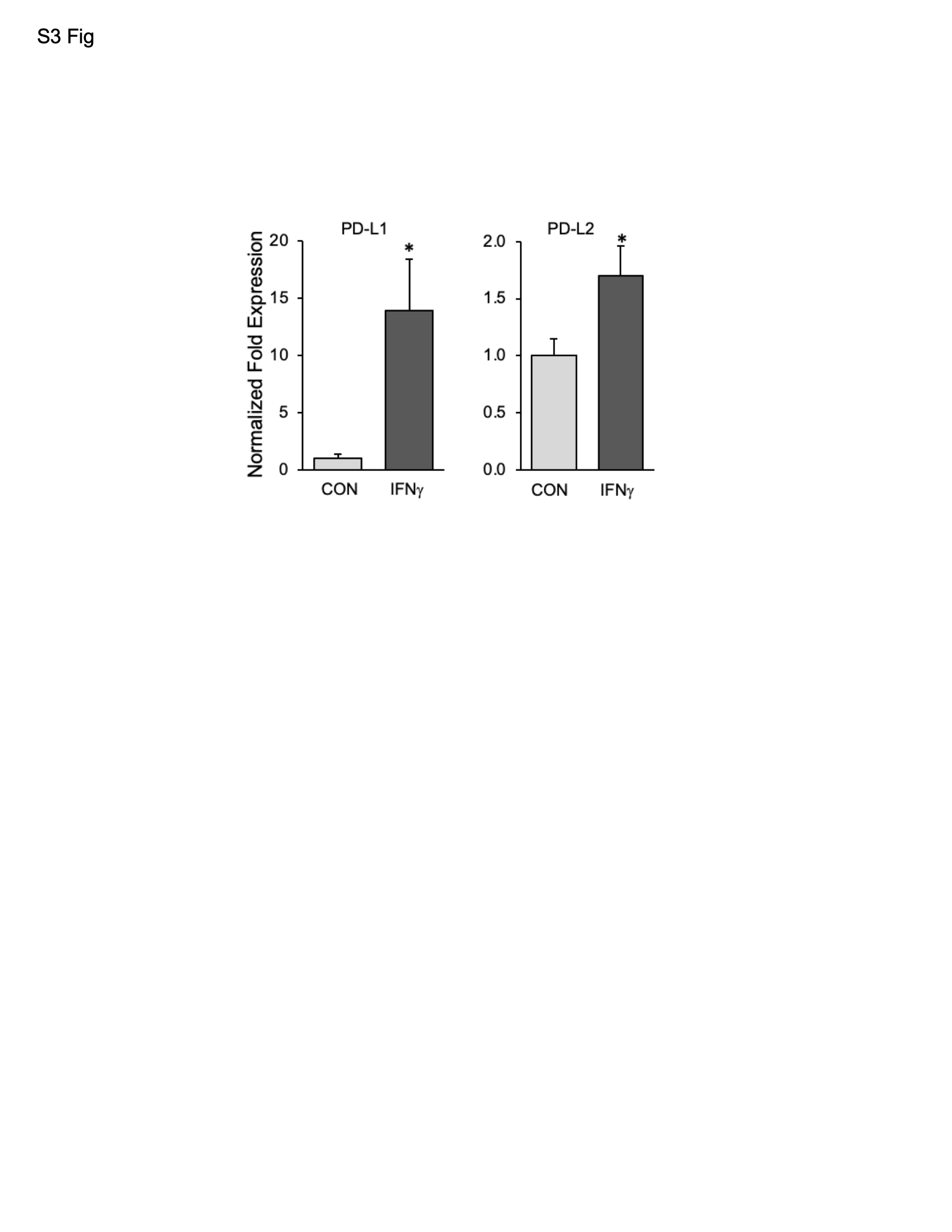

Supplement: S3 Fig — The mRNA level of PD-L1 and PD-L2 was measured by qRT-PCR and normalized to GAPDH in BE(2)-C cells treated with IFNγ at 10 ng/ml for 24 hours. (TIF) [file pone.0277956.s003.tif]

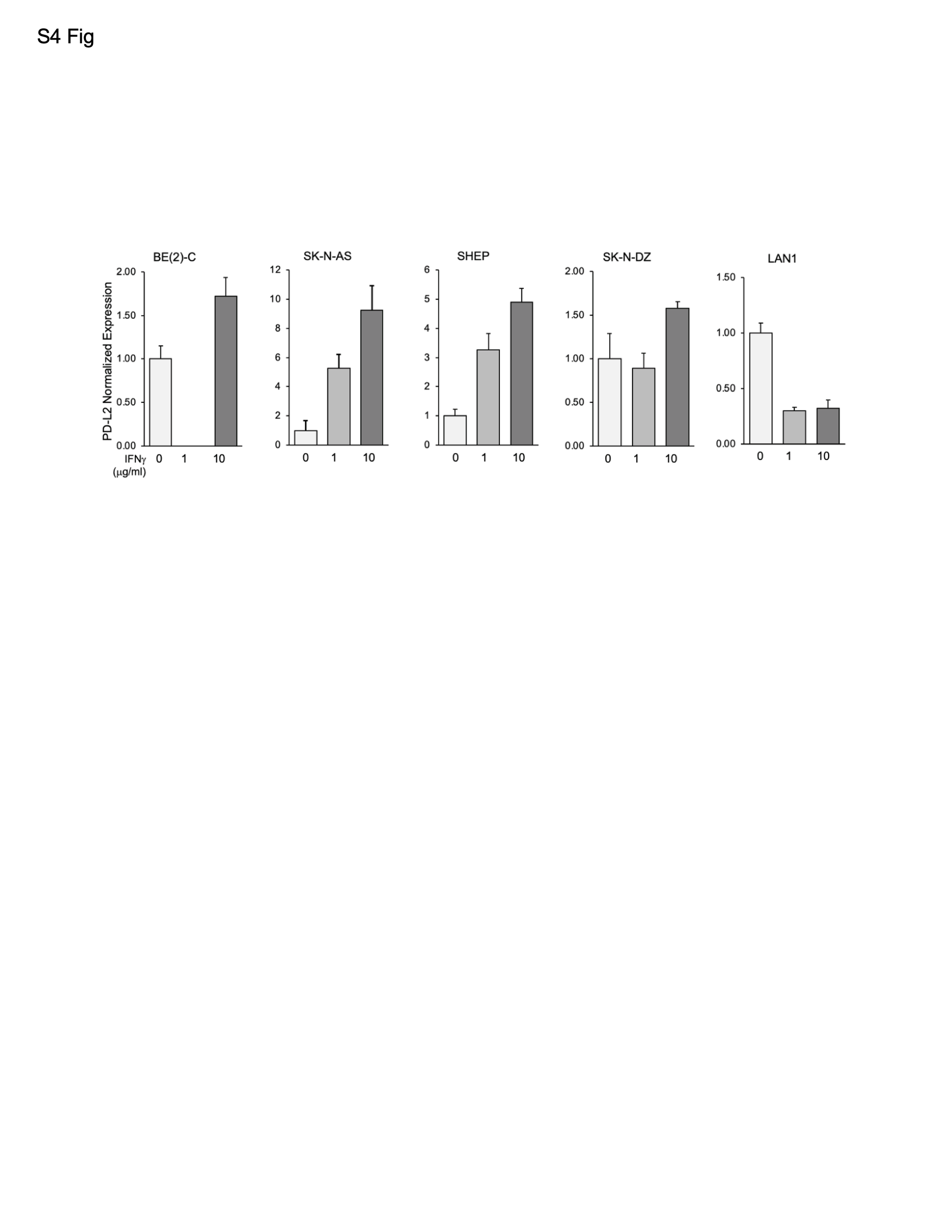

Supplement: S4 Fig — The mRNA level of PD-L2 was measured by qRT-PCR and normalized to GAPDH in five human neuroblastoma cell lines. All cells were treated with IFNγ at 0, 1, or 10 ng/ml for 24 hours. (TIF) [file pone.0277956.s004.tif]
