# Supplementary material for: Anti-GRP-R monoclonal antibody antitumor therapy against neuroblastoma
Source: PLoS One. 2022 Dec 16;17(12):e0277956. doi: 10.1371/journal.pone.0277956 (PMC9757561; doi:10.1371/journal.pone.0277956)

Fig 2_raw_images

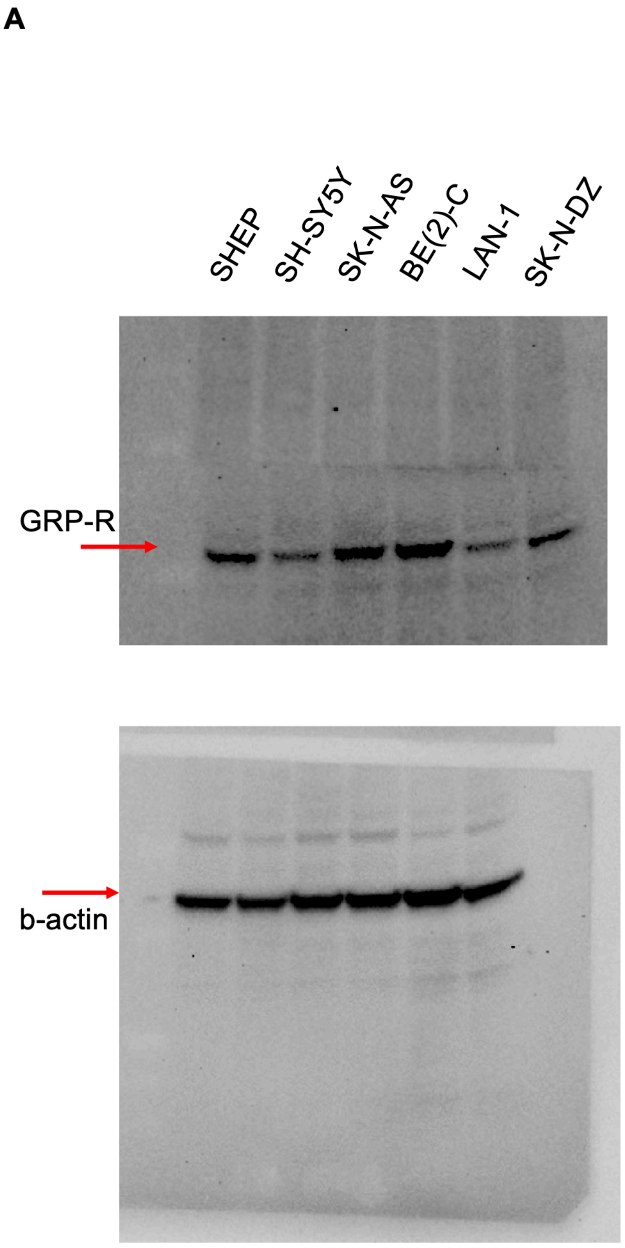

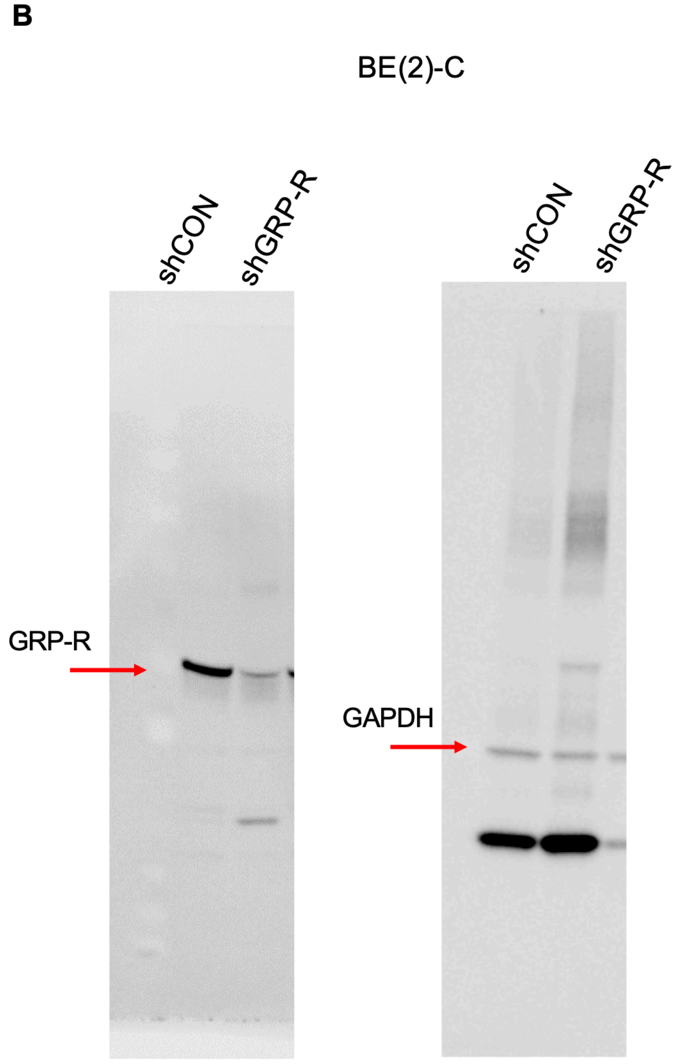

Fig 3_raw_images

A

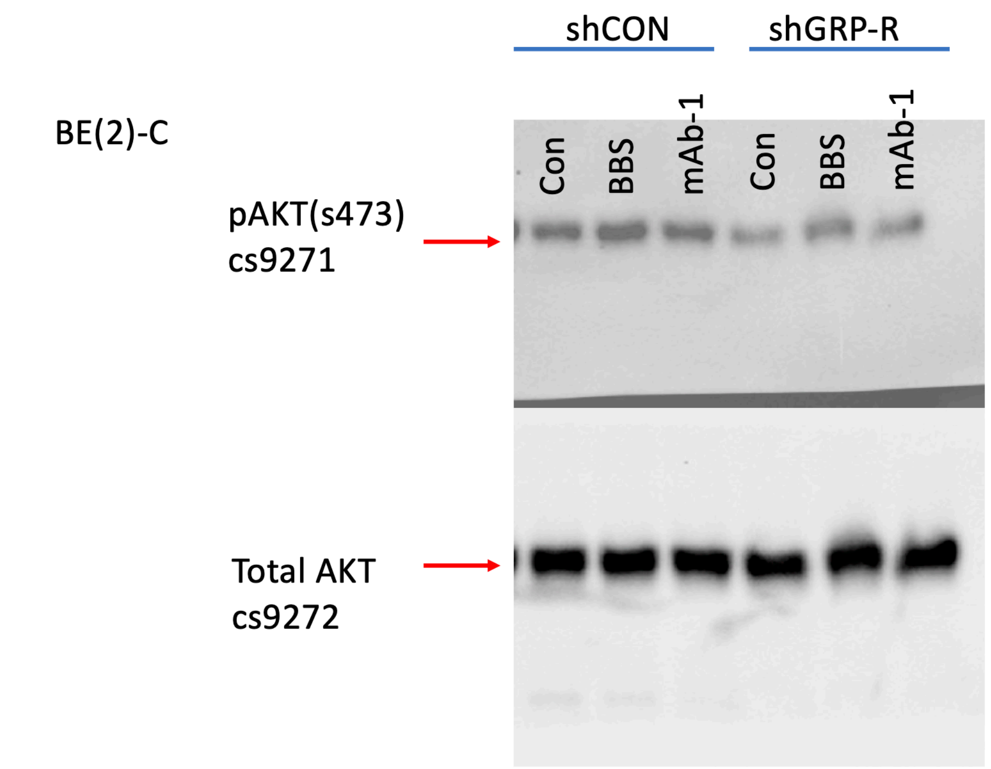

B

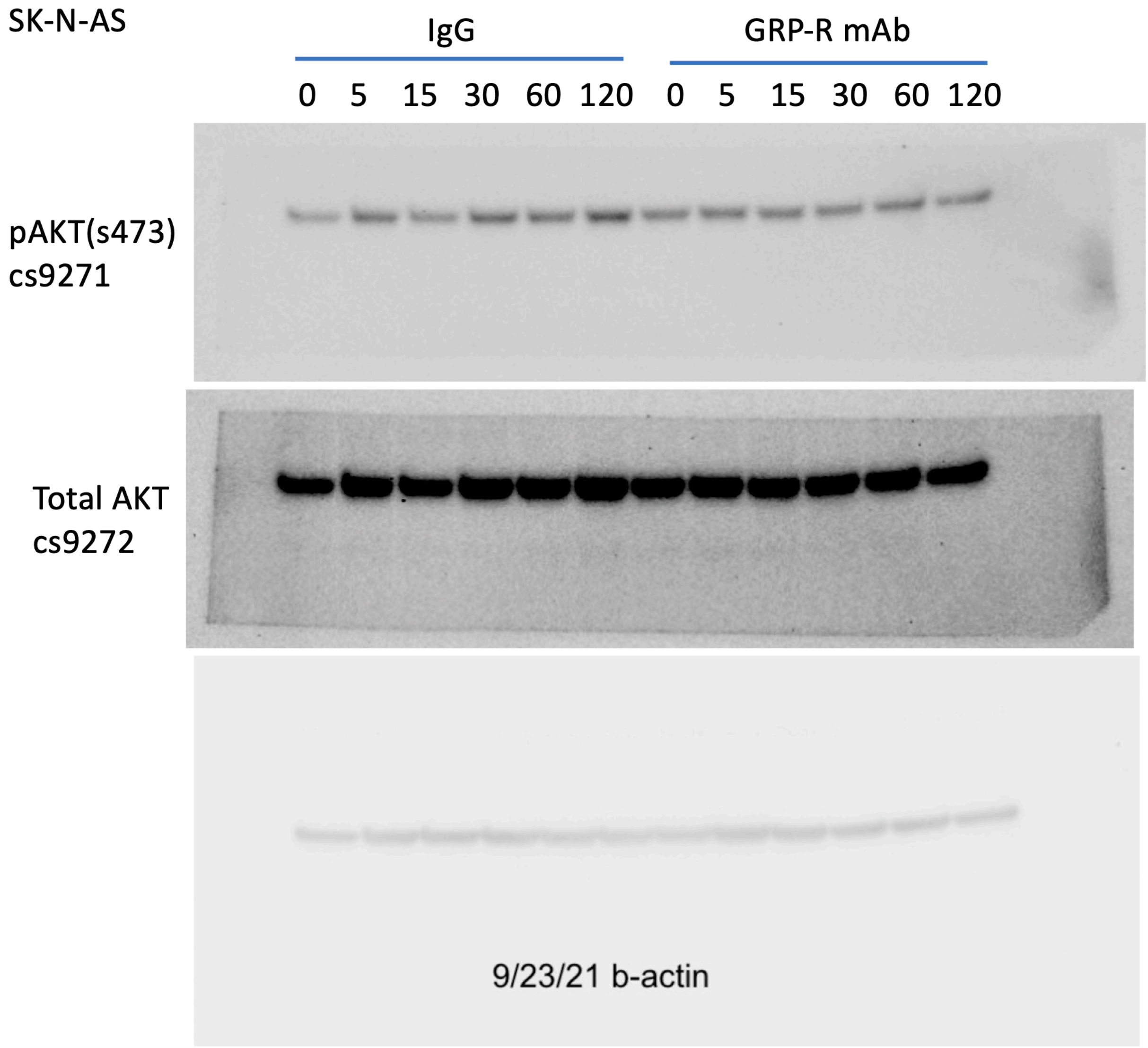

Fig 5_ raw_images

B

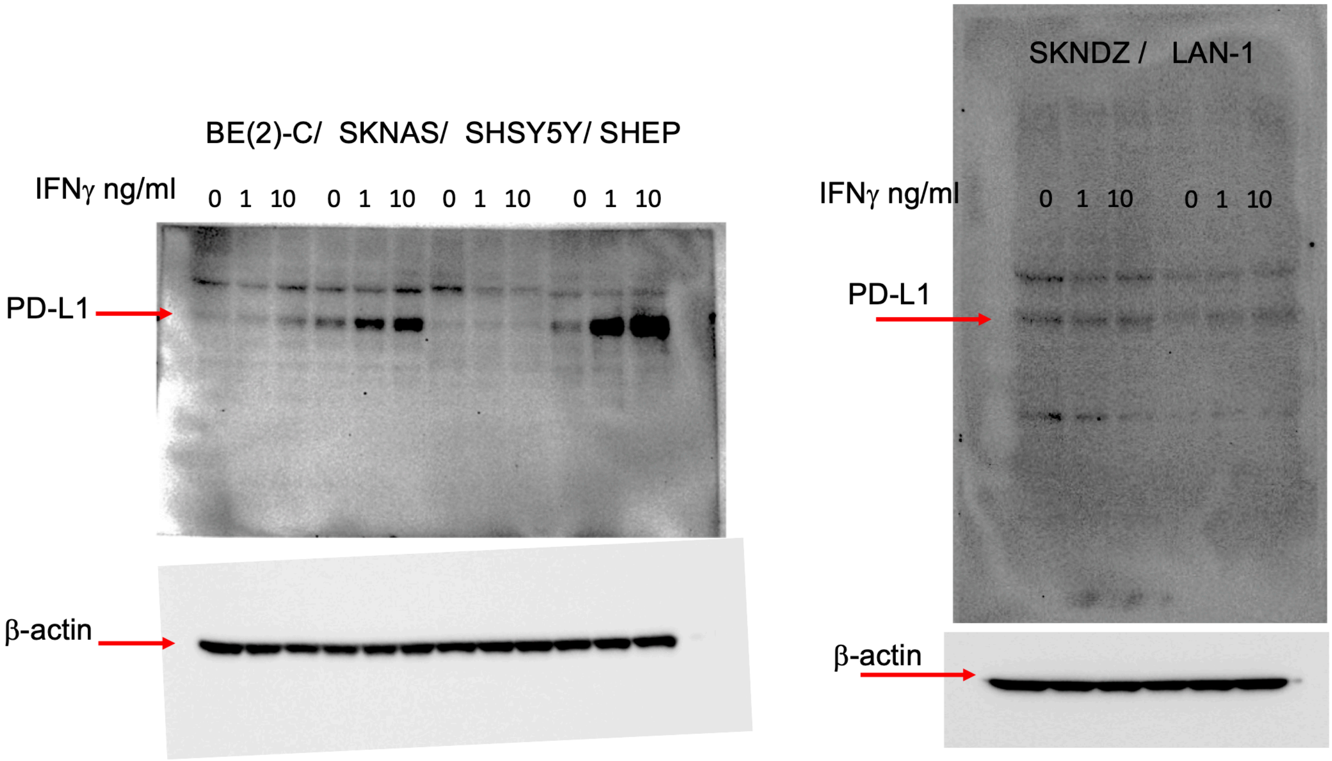

Fig 5D_ raw_images

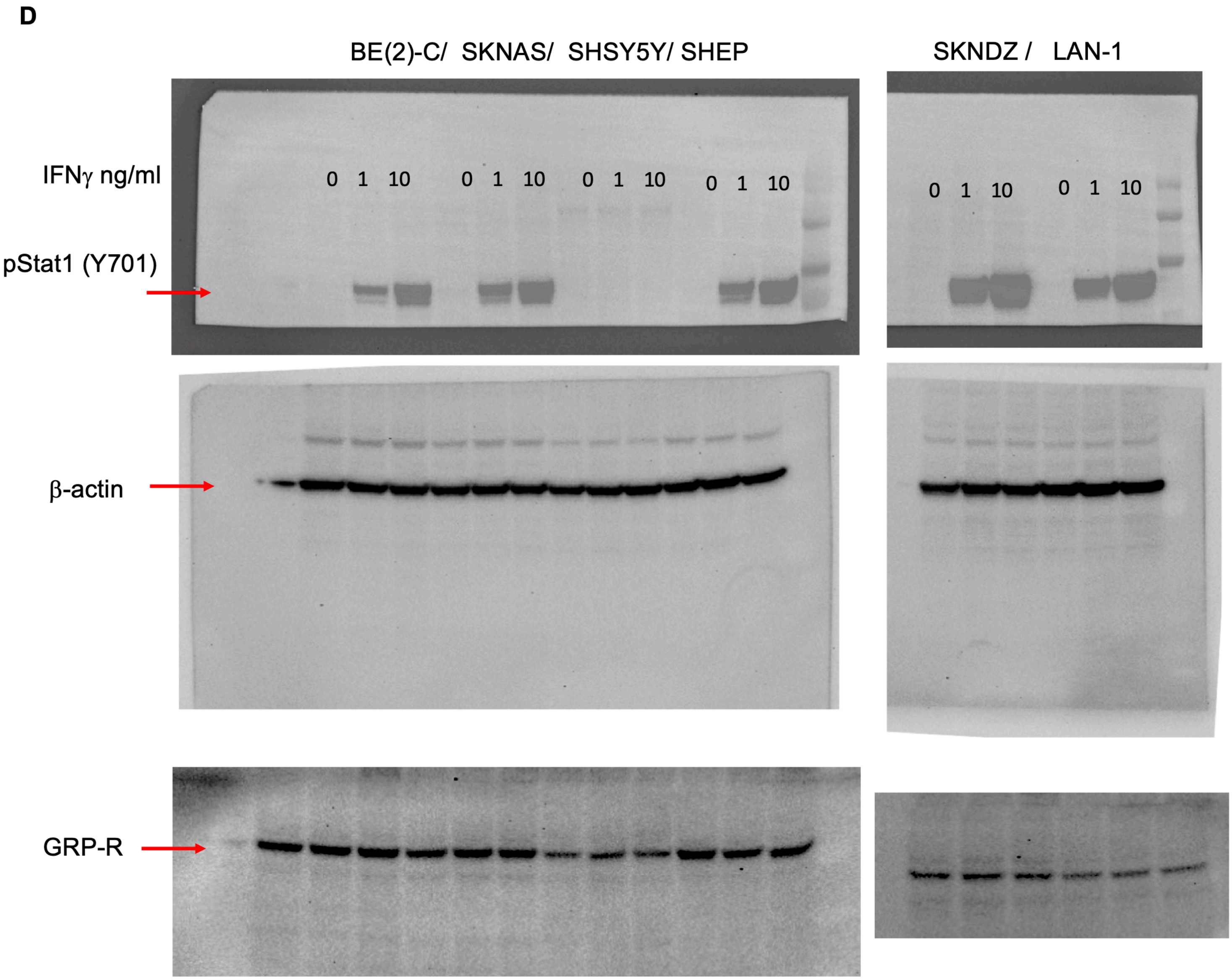

Supplement: S1 Raw images — (PDF) [file pone.0277956.s005.pdf]
