# Supplementary material for: Anti-GRP-R monoclonal antibody antitumor therapy against neuroblastoma
Source: PLoS One. 2022 Dec 16;17(12):e0277956. doi: 10.1371/journal.pone.0277956 (PMC9757561; doi:10.1371/journal.pone.0277956)
Supplement: Attachment — Submitted filename: Response to Reviewers_100722.dc edits.pdf [file pone.0277956.s006.pdf]

**Dai H. Chung, M.D.**
Professor and Strauss Chair in Pediatric Surgery
Executive Vice Chair, Department of Surgery
Chief Medical Officer, Joint Pediatric Enterprise

October 7, 2022

*Leonidas G Koniaris M.D.*
*Board Editor*
*PLOS ONE*

RE: Manuscript [PONE-D-22-10312]

Dear Dr. Koniaris,

Thank you for considering our manuscript entitled "Anti-GRP-R monoclonal antibody antitumor therapy against neuroblastoma" for publication in *PLOS ONE.* We appreciate the insightful comments and suggestions by editor and Reviewers. As requested, we have updated our changes and additions in a point-by-point manner below:

**Reviewer Comments:**

**Reviewer #1:**

*1. P 5, top: How does the statement about N-803 relate to this work?*

We thank the reviewer's comments. N-803 is not particularly related to this work; therefore, the statement "N-803, an IL-15 super agonist" was deleted.

2. *P 14, middle: How many PDXs were evaluated? What was the breakdown of risk-category of the patients from which these PDXs were derived?*

We obtained four PDXs from the Children's Oncology Group (COG). All PDXs were established using samples from patients with stage IV disease. COG-N-619x was derived from metastatic tumor cells in bone marrow and *MYCN*-non-amplified (32 months at age of diagnosis and therefore high risk). COG-N-415x and COG-N-440x were derived from metastatic tumor cells in blood and *MYCN*-amplified (high risk). COG-N-496x was derived from metastatic tumor cells in bone marrow, and *MYCN*-amplified (high risk).

We stained all four PDXs. GRP-R was positive with GRP-R mAb-1 staining. We present the stained samples of PDX COG-619 in Figure 2E (top) as representative examples of positive staining for GRP-R versus secondary antibody only.

*3. P 16, "This suggests that the immune checkpoint pathway…" What data presented demonstrates that activation of these immune checkpoint pathways suppressed the measured ADCC? If this is speculation, then this statement should be confined to the discussion.*

We agree with the reviewer's comments. We deleted the sentence "This suggests that the immune checkpoint pathway…" from the results section on page 16.

*4. P 18, bottom: Do these data that "there was no difference in tumors treated with GRP-R mAb alone" suggest the only mechanism of antitumor effects of this antibody are through ADCC? Please comment.*

In this study, we initiated the treatment with visible tumors at a diameter of approximately 7 mm using GRP-R antibody at the dose of 4 mg/kg. Mice were treated twice per week for a total of 3 weeks. The total treatment protocol was only six doses. Furthermore, the tumor xenografts were established with aggressive BE(2)-C cells. Tumor growth was very rapid and antibody treatment alone was insufficient to inhibit tumor growth, although we demonstrated that GRP-R mAb treatment resulted in the inhibition of PI3K/AKT activity downstream of GRP/GRP-R. In the current conditions, the antitumor effects of antibody treatment appear to be mediated via ADCC. However, it is possible that earlier or more prolonged antibody treatment, and/or treatment of a less aggressive tumor cell line would have facilitated greater efficacy with mAb treatment alone. We hypothesize that earlier treatment or a longer duration of GRP-R mAb treatment could potentially lead to greater inhibition of tumor growth with GRP-R mAb treatment alone.

*5. P 19, first paragraph: "highly effective treatment strategy". Is this not somewhat of an overstatement? Please comment.*

We agree with the reviewer's comments. We revised our statement to *"We demonstrate a novel treatment strategy using GRP-R mAb against neuroblastoma."* The words "*and highly effective*" was deleted.

*6. P 20, bottom: How did you demonstrate that the decrease in ADCC at 24 hours was a direct result of activation of the PD-1/PD-L1 pathway? Please clarify.*

We agree with the reviewer's comments. We revised the sentence to *"However, we found that GRP-R mAb-mediated induction of ADCC was inhibited at 24 hours compared to 4 hours, suggesting that the action of ADCC can be inhibited over time due to NK cell dysfunction (Judge, SJ. Et al. 2020)."*

*7. P 22, top and references 48 and 49: I agree that anti-GRP mAb would benefit from clinical testing but it is unclear to me how these two studies suggest using an anti-GRP mAb would be a "highly effective immunotherapeutic strategy". Please comment.*

We agree with the reviewer's comments. We have revised the statement to *"…suggests GRP-R mAb is a potentially novel immunotherapeutic treatment against neuroblastoma that would benefit from clinical testing."*

*8. P 23, last paragraph, also see comment #4 above: do these antibodies inhibit NB tumorigenesis? Please comment.*

GRP-R mAbs inhibit tumorigenesis via inactivation PI3K by decreasing cell growth, inhibiting colony formation (Figure 3), and via ADCC by promoting cell death, as demonstrated by increased cell lysis (Figure 4) *in vitro*, as well as preventing cell division and mitosis *in vivo* (Figure 6).

We have previously established that inhibition of GRP binding with GRP-R attenuates neuroblastoma tumorigenesis through multiple mechanisms, including inhibition of the PI3K-AKT pathway, and we believe that GRP-R mAbs could potentially serve as a novel immunotherapeutic strategy in humans with potentially lower treatment morbidity and toxicity than standard chemotherapy.

*9. P 23, last sentence: The use of "will" seems too definitive for the data presented?*

We appreciate the reviewer's comment and have changed "*will*" to "*may*".

**Reviewer #2:**

*Major points*

*1. Current figures should be replaced with higher res ones.*

We replaced the figures with higher resolution images.

*2. The authors described that the affinity of antibodies was measured by Octet RED96. However, we did not find any data to support this.*

We appreciate the reviewer's comments. These data, including GRP-R mAb $K_D$ values, have been added as Supplemental Figure 1. GRP-R mAb-1 had the lowest $K_D$ value and the highest affinity.

*Minor points*

*1. Consider not to use "the highest affinity" in the Abstract, detailed EC50/KD values was better. Similarly, "The cytotoxic effects of mAb-1 were confirmed in vivo using a murine tumor xenograft model", please make a conclusion with detailed result.*

We thank the reviewer for these comments. We have revised the sentence to "*GRP-R mAb-1 demonstrated the highest efficacy with the lowest $EC_{50}$ at 4.607 ng/ml*". *"The cytotoxic effects of mAb-1 were confirmed with secretion of cytotoxic granzyme B from NK cells and reduction of mitotic tumor cells in vivo using a murine tumor xenograft model."*

*2. The affinity of an antibody does not equal to EC50. It is said that "GRP-R mAb-1 has the highest affinity with the lowest EC50 at 4.607 ng/ml", I recommend to mention something instead of it.*

We corrected the sentence to "*GRP-R mAb-1 has the greatest efficacy with the lowest $EC_{50}$ at 4.607 ng/ml.*"

*3. It is said that "Four peptides of the GRP-R extracellular domains (E1, E2, E3, and E4) were synthesized and used to screen the phage library for scFv binders" and only four mAbs against E1 were obtained. What's the panning result of scFv binders against E2/E3/E4? Do the four antibodies have the cross reactivity to E2/E3/E4? And we do not see any control peptide/protein in Figure 1D.*

While performing this study, we obtained four mAbs against E1, which we believe were more than sufficient to examine our hypothesis. When we tested the specificity of GRP-R mAbs to GRP-R extracellular domain E1, we indeed examined the cross reactivity to other three GRP-R extracellular domains E2, E3, and E4 and found that there was no binding signal detected, indicating GRP-R mAbs do not demonstrate cross reactivity to E2, E3, and E4. The results were added as Supplementary Figure 2.

*4. Scale bar is missing in Figure 2E*

We apologize for this oversight. The scale bar has been added.

Children's Health, 1935 Medical District Drive, Mailstop F3.66 | Dallas, TX 75235 | Ph: 214-456-6040 | Fax: 214-456-6320

*5. What does abbreviation BBS stand for? It's missing.*

We apologize for this oversight. BBS stands for bombesin. We added the abbreviation "*BBS*" after bombesin on page 7.

*6. Some typos. For example, IFNg should be IFN-γ; "CD3-CD56+" , the "-" and "+" need to be superscripted; "Alex 488" should be "Alexa Fluor 488", et. al.*

We thank the reviewer's comments. We have revised the typos in the manuscript.

**<u>Additional Edits:</u>**

1. All immunoblot raw images were submitted as "Figs_raw_images".

2. In addition, we added more detailed information regarding our animal studies in the methods section, including basic housing, method of sacrifice, method of anesthesia and/or analgesia, and humane endpoint.

3. Funding Statement:

This work was supported by a grant from the National Institutes of Health (R01 DK61470). This work was supported in part by the Welch Foundation grant AU-0042-20030616 (ZA), and Cancer Prevention and Research Institute of Texas (CPRIT) Grants RP150230, RP150551, and RP190561 (ZA). The funders had no role in study design, data collection and analysis, decision to publish, or preparation of the manuscript.

We have addressed all concerns and incorporated changes in this revised manuscript. We hope that your Editorial Board will find our revised manuscript suitable for publication in *PLOS ONE*. We appreciate you for reconsidering our manuscript and look forward to hearing back from you.

Sincerely,

Dai H. Chung, M.D.
